# Clonal dominance of CD133^+^ subset population as risk factor in tumor progression and disease recurrence of human cutaneous melanoma

**DOI:** 10.3892/ijo.2012.1590

**Published:** 2012-08-17

**Authors:** BHUVNESH K. SHARMA, V. MANGLIK, MICHAEL O’CONNELL, ASHANI WEERARATNA, EDWARD C. McCARRON, JENNIFER N. BROUSSARD, KYLE A. DiVITO, CYNTHIA M. SIMBULAN-ROSENTHAL, DEAN S. ROSENTHAL, JOHN L. ZAPAS

**Affiliations:** 1 Department of Surgical Oncology, The Maryland Melanoma Center, The Harry and Jeanette Weinberg Cancer Institute; 2 Department of Pathology, MedStar Franklin Square Medical Center, Baltimore, MD;; 3 Elizabeth City State University, Elizabeth City, NC;; 4 Department of Biochemistry and Molecular and Cellular Biology, Georgetown University, School of Medicine, Washington, DC;; 5 Molecular and Cellular Oncogenesis Program, The Wistar Institute, Philadelphia, PA 19104, USA

**Keywords:** CD133, human melanoma, disease recurrence

## Abstract

Chemotherapeutic refractoriness of advanced cutaneous melanoma may be linked with melanoma-initiating cells, also known as melanoma stem cells. This study aimed to determine relative risk of clonal dominance of the CD133^+^ phenotype in tissues from melanoma patients with different clinical outcomes that could be applied to early diagnosis, prognosis or disease monitoring. Significant overexpression of CD133 (p<0.02) was observed by immunohistochemical staining in tissues from patients with recurrent disease versus those without disease recurrence. Relative risk analysis between these two groups suggested that the patients with recurrence or metastatic lesion had a greater than 2-fold overexpression of CD133. In addition, immunodetectable CD133 corroborated with upregulation of CD133 RNA levels (14- to 30-fold) as assessed by quantitative real-time reverse transcription-PCR (qRT-PCR) comparison of melanoma cell lines derived from patients with poor clinical outcomes and short overall survival (<10 months), vs. those derived from patients with good clinical outcomes and longer overall survival (>24 months). Further, cells derived from patients, and MACS-sorted according to their CD133 status retained their CD133-positivity (>95%) or CD133-negativity (>95%) for more than 8 passages in culture. CD133^+^ cells could repopulate and form tumors (p<0.03) in athymic *NCr-nu/nu* mice within 8 weeks while no tumors were observed with CD133^−^ phenotype (up to 200,000 cells). Taken together, the study demonstrates, for the first time, that there exists a clonal dominance of a CD133^+^ population within the hierarchy of cells in cutaneous tissues from patients that have undergone successive progressive stages of melanoma, from primary to metastatic lesions. CD133, thus, provides a predictive marker of disease as well as a potential therapeutic target of high-risk melanoma.

## Introduction

High-risk cutaneous melanoma, a highly aggressive form of skin cancer, is minimally susceptible to current treatment strategies for systemic disease and thus these strategies have not yielded any appreciable survival benefit. Chemotherapeutic refractoriness of advanced cutaneous melanoma is attributable to several resistance mechanisms resulting from the outgrowth of resistant clones, subsequently promoting tumor recurrence and metastasis. Recently, therapy-resistant clones are implicated as melanoma initiating cells (MICs) also known as melanoma stem cells (MSCs). CD133 is now recognized as an important cancer stem cell-associated marker in cutaneous melanoma.

The existence of cancer cells with stem cell-like characteristics has been reported in a variety of human malignancies (1–5) including melanoma (6–10). The identification and characterization of stem cells in normal tissue has expedited isolation of cancer stem cells (CSC) and provided biomarkers for identification of a candidate CSC population (11). In addition, the prospective isolation of CSC and comparison to normal stem cells/progenitors sheds light on the origin, regulation and association with disease pathogenesis attributable to CSCs (12). The variability in frequency of CSCs within the tumor and the existence of tumor-specific stem cell markers has been a subject of controversy.

Several reports have implicated the expression of different markers of melanoma stem cells, including the self-renewal transcriptional factor, Bmi-1, in primary and metastatic melanoma. Four subpopulations of cells have been identified in melanoma tissues, including CD20-positive, CD133-positive, label-retaining cells and side-population (SP) cells. These cell populations possess stem-cell characteristics with defined self-renewal capacity, differentiation potential, and high tumorigenicity (13). In addition, ABCB5 chemoresistance mediator (14), CD166 activated leukocyte cell adhesion molecule (15), and Nestin intermediate filament protein expressed in the cytoplasm of intraepithelial stem cells (16), have also been reported in various stages of cutaneous melanoma (9,10). Although CD133 has recently been recognized as a potential stem cell marker capable of identifying a tumor-initiating population in solid tumors, the consistent overexpression of CD133 cells in conjunction with other melanoma stem cell markers among patients with high-risk primary or recurrent melanoma has not yet been clearly established and no preferential CD133 ligands or connections with signaling pathways have been elucidated.

The present study examines relative risk analysis on clonal dominance of CD133^+^ MSCs in tissues that could be applied to early diagnosis, prognosis, or to the development of more effective strategies have or disease monitoring and treatment modalities. The expression levels of CD133 were thus assessed in tissues from patients with or without recurrent disease and compared with melanoma thickness as a prognostic factor. In addition, CD133 expression in tissues from patients with primary melanoma successively progressed to lymph node metastasis and distant metastasis were compared, utilizing immunohistochemistry (IHC) and quantitative real-time reverse transcription-PCR (qRT-PCR).

## Materials and methods

### Immunohistochemical staining of CD133, ABCB5, CD166 and Nestin in melanoma tissue sections

Formalin-fixed, paraffin-embedded (FFPE) tissues were selected from 3 groups of consenting patients, after Institutional Review Board protocol approval. Group I consisted of melanoma patients who did not develop disease recurrence or metastases (n=18). Group II was comprised of melanoma patients who developed metastatic lesions (n=18). Group III included patients with disease progression from primary melanoma to lymph node (LN) metastasis and subsequently to distant metastasis (n=8). Tissue sections measuring 5 *μ*m were subjected to immunohistochemical staining using monoclonal antibodies to a distinct subset of MSCs including CD133/prominin-1 (Abcam), a CSC and neural stem cell marker; ABCB5 (Genway), a chemoresistance mediator; CD166 (BD Biosciences), an adhesion molecule; and Nestin (Abcam); a lineage marker. The tissue sections were immunostained using a Bond-Max automated staining system (Leica). The reactions were developed using a biotin-free bond polymer refine detection kit (Leica) and visualized with 3′3-diaminobenzidine (DAB) substrate. Immunoexpression of stem cell markers was evaluated with a Zeiss Axioscope microscope using a semi-quantitative scoring system for intensity of staining of stem cell markers on tumor cells. Fisher’s exact test, Odds ratio analysis and relative risk analysis were performed to determine statistical significance.

### Establishment and characterization of human melanoma cell lines

Human melanoma cell lines were established from fresh metastatic tumor tissues of consenting patients comprising two groups. Group A (n=4) consisted of patients (FS-4, FS-5, FS-7 and FS-9) with poor clinical outcomes and short overall survival (<10 months), and group B (n=4) was comprised of melanoma patients (FS-11, FS-12, FS-13 and FS-14) with good clinical outcomes and longer overall survival (24 months). The majority of cell lines were derived from lymph node metastases.

Single cell suspensions were prepared from freshly resected tumor tissue specimens by mechanical mincing; no enzymatic dissociation was used. Viable tumor cells were cultured in Iscove’s medium supplemented with 10% fetal bovine serum (FBS) and antibiotics. After overnight incubation at 37°C with 5% CO_2_, floating debris was discarded and fresh complete medium was added. Cultures were fed 2–3 times per week, replacing about half of the spent medium. Melanoma cell lines were split when near confluence and sub-cultured at 4×10^4^ viable cells per cm^2^ surface area in polystyrene culture flasks. Cultures were shown to be free of mycoplasma contamination using the MycoProbe™ mycoplasma detection kit (R&D Systems, Minneapolis, MN, USA). To insure that each cultured cell line consisted of melanoma cells, each cell line was stained and analyzed by flow cytometry for melanoma-specific antigens MART-1, gp100, TRP75, or melanoma-associated chondroitin sulfate proteoglycan (MCSP). At least one of these four melanoma antigens was observed in order to include the cell line in the study. All cell lines were early passages of less than 20.

### Isolation of CD133^+^ and CD133^−^ subsets of MSCs from early passages (<20) of cell cultures of metastatic melanoma

CD133^+^ or CD133^−^ cells were isolated from cell culture suspensions per kit instructions using magnetically labeled CD133 Micro Beads and a MACS^®^ Column (Miltenyi Biotech, Auburn, CA). To increase the purity, the positively selected cell fraction containing the CD133^+^ cells were further separated over a second MACS^®^ column.

### In vivo assessment of CD133^+^ vs. CD133^−^ tumorigenicity in athymic NCr-nu/nu mouse xenotransplantation assay

CD133^+^ or CD133^−^ cells were cultured in DMEM/F-12 (1:1) plus 1% penicillin/streptomycin, 20 *μ*g/ml each of epidermal growth factor (EGF) and fibroblast growth factor (FGF), and 1X B-27 supplement (Invitrogen). Culture plates were coated in 10 mg/ml 2-hydroxyethly methacrylate (polyHEMA; Sigma, St. Louis, MO) in order to ensure a spheroid suspension. Flow cytometry was used to confirm the populations as CD133^+^ or CD133^−^ using the monospecific mouse mAb CD133/2 (Miltenyi Biotech). Athymic *NCr-nu/nu* mice were purchased from Harlan Laboratories (IN). Purified CD133 cells were counted using a Coulter Counter (Beckman Coulter Brea, CA) and then serially diluted. Cells were re-suspended in a mixture of growth media containing Matrigel (BD, Bedford, MA), and injected subcutaneously into each hind flank. Animals were each injected with 2×10^5^ or 1×10^3^ of either CD133^+^ or CD133^−^ cells; each group of cells was injected in replicates of four. Animals were allowed to recover from anesthesia and monitored daily to check for the presence of palpable tumors. Tumor growth was measured using a digital micrometer and calculated using the modified ellipsoid formula (1/2 length x width^2^) for tumor volume as described previously (17,18). Tumor volumes were recorded each week for a period of eight weeks. All animal studies were performed in compliance of regulations of Georgetown University Animal Care and Use Committee (GUACUC; Protocol 10-051; expiration 10/18/2013) Washington DC, USA.

### CD133 gene transcripts by qRT-PCR

Total RNA was prepared from 8 melanoma cell lines established as described above using Trizol (Invitrogen) and an RNeasy Mini Kit (Qiagen). From 1 *μ*g RNA, cDNA was synthesized using an iScript™ cDNA synthesis kit (Bio-Rad) according to the manufacturer’s instructions. Absolute quantitation of CD133 gene transcripts was performed using qRT-PCR analysis on a 7500 real-time PCR system using the SYBR Green method (Applied BioSystems). The following human-specific intron spanning primer pairs for CD133 were used: forward, CATCCACAGATGCTCCTAAGGC; reverse, GCTTTATGGGAGTCTTGGGTC. Cycle conditions were as follows: 1 cycle at 95°C for 10 min, 40 cycles at 95°C for 15 sec, and 60°C for 1 min. The specificity of the PCR product was verified by melting curve analysis. Threshold cycles of primer probes were normalized to those of 18S rRNA. Absolute values of transcripts were calculated using the standard curve method.

## Results

### Immunohistochemical expression of MSCs

**Figure 1.**
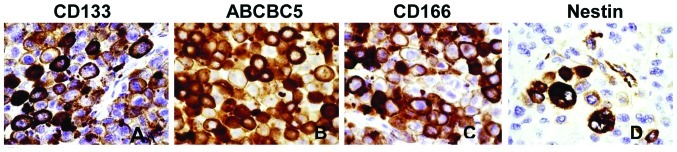
Immunohistochemical expression of CD133, ABCB5, CD166 and Nestin (A, B, C and D respectively) in FFPE tissues from primary melanoma. FFPE tissues were deparaffinized, and immunostained using a Bond-Max automated staining system (Leica). The reactions were developed using a biotin-free bond polymer refine detection kit (Leica) and visualized with 3′3-diaminobenzidine (DAB) substrate. Among positive immunostained tissues of primary melanomas, staining tended to be focal with a spectrum ranging from moderate-to-strong (original magnification x40).

As shown in Fig. 1, CD133 and CD166 specific mAb stained the membrane as well as the cytoplasm to a lesser degree, while Nestin and ABCB5 mAbs predominantly stained cytoplasm of MSCs. Tables I–III summarize the IHC expression levels of CD133, ABCB5, CD166 and Nestin in tissues derived from patients with different clinical outcomes.

### Melanoma patients with disease recurrence or non-recurrence

Significantly greater expression of CD133 in tissues was seen in melanoma patients who developed metastatic lesions/recurrence of the disease, when compared with the group of melanoma patients who did not develop any metastatic lesions or recurrence (p<0.02, Fisher’s exact test; Table I). This significant difference was also confirmed by Odds ratio analysis (p=0.025). Further, relative risk analysis of these two groups of melanoma patients suggested CD133-immunopositivity was 2-fold higher in tissues from patients with recurrence or metastasis. No significant differences in expression levels of ABCB5, CD166 or Nestin were observed in these groups.

### Primary melanocytic lesions with different thickness

Comparison of immunoexpression of CD133, ABCB5, CD166 and Nestin in tissues with different primary lesion thicknesses revealed greater expression of CD133 and CD166 in primary melanomas that demonstrated >1.00 mm thickness compared to those that were less than ≤1.00 mm. However, this difference was not statistically significant (p>0.05; Table II).

### Patients with primary melanoma successively progressed to lymph node metastasis and distant metastasis

Consistent high levels of CD133 and ABCB5 marker expression were observed in 100% tissues from patients with primary melanoma that successively progressed to lymph node metastasis (100%) and distant metastasis (83% and 66%, respectively; Table III). The levels and incidence of CD166 expression was low in all stages of disease and also not altered significantly (p>0.05). Nestin expression levels were found to be conserved in all tissues of the study groups (100%). However, no statistical significance (p>0.05) was observed in the overexpression of CD133 or ABCB5 in this study group.

### Expression of CD133^+^ mRNA transcripts in cell cultures established from tissues of patients with poor outcome and short overall survival, good outcome and long overall survival

**Figure 2.**
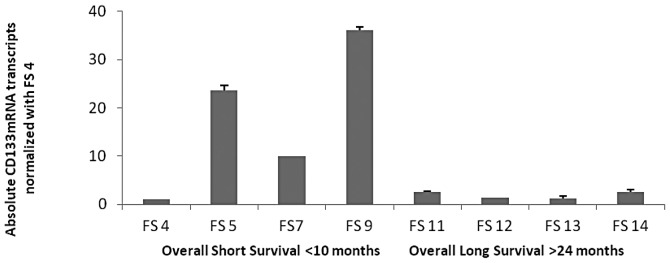
CD133 transcripts in low passage cell lines from patients with short survival (<10 months) compared to those with long overall survival (>24 months; p<0.05). CD133 RNA was measured by qRT-PCR: total RNA was prepared from the melanoma cell lines established as described in Materials and methods. Threshold cycles of primer probes were normalized to those of 18S rRNA. Absolute values of transcripts were calculated using the standard curve method. Each set of experiments was conducted in duplicate and error bars depict standard deviation from mean absolute values of transcripts derived from each independent experiment.

To investigate the potential of CD133 as a molecular biomarker of melanoma progression and disease recurrence, we assessed CD133 mRNA transcript levels by qRT-PCR in low passage (<20) primary cell lines established from fresh tissues of patients with either poor outcome and short overall survival, or from patients with good outcome and long overall survival status. Using 18S rRNA as a control, CD133 mRNA expression levels in each individual cell line derived from patients with poor clinical outcome and short overall survival (FS-4, FS-5, FS-7 and FS-9) were compared with four cell lines derived from melanoma patients with good clinical outcome and longer overall survival (FS-11, FS-12, FS-13, FS-14; Fig. 2). CD133^+^ mRNA transcripts were expressed at levels 15–30 times higher in the latter group, suggesting that CD133 transcripts strongly and negatively correlated with the clinical outcome, and were thus a potential predictor of poor prognosis in high-risk melanoma (p<0.04).

### In-vivo tumorigenicity of CD133^+^ and CD133^−^ phenotypes in xenotransplantation in athymic NCr-nu/nu mice

**Figure 3.**
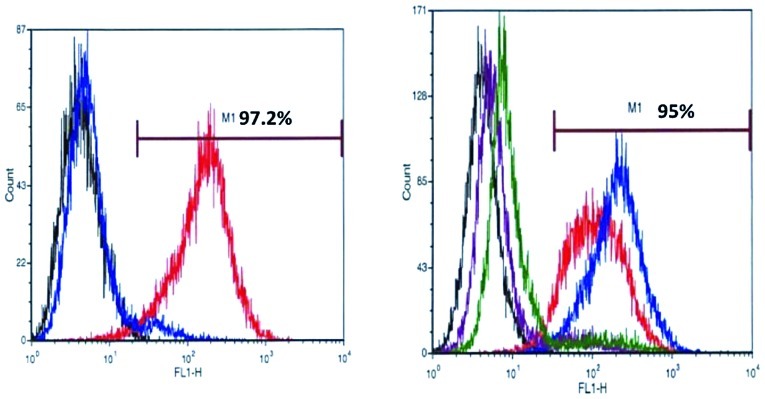
Melanoma cells maintain consistent expression levels of CD133 in culture. Melanoma cells were sorted for CD133 twice by MACS, cultured in suspension for either two (A) or eight (B) passages, then analyzed for CD133 expression, as described in Materials and methods. A, Melanoma cells passaged two times in culture were analyzed for the presence or absence of CD133 by flow cytometry. The CD133^+^ passage 2 culture is depicted by the red line (M1) by flow cytometry, and are 97.2% CD133^+^. Cells originally sorted from the same population for the absence of CD133, then passaged twice in culture are shown by the blue line, and are 95% CD133^−^. The black line shows the same melanoma cells without anti-CD133 antibody as a negative control. B, Cells passaged eight times in culture were analyzed as in (A). Red and blue lines represent two different CD133^+^ cultures (95% positive), while purple and green lines represent two CD133^−^ cultures (95% negative).

**Figure 4.**
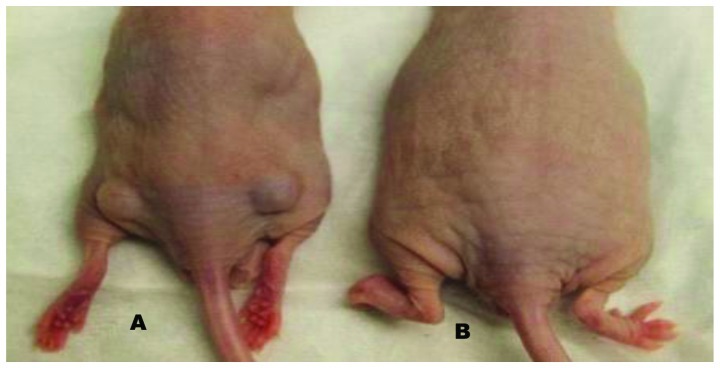

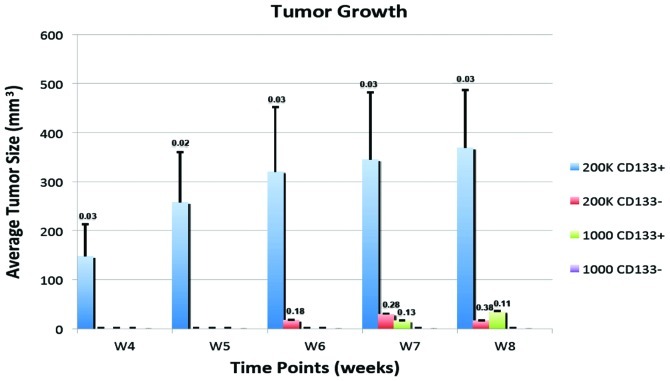
Only CD133^+^ cells form tumors in athymic nude mice. Purified CD133 cells were counted, serially diluted, resuspended in a mixture of growth medium containing Matrigel, and subsequently injected subcutaneously into each hind flank. A, Animals were injected with 1000 CD133^+^ cells. B, Animals were injected with 1000 CD133^−^ cells. Tumor sizes from each group (n=4) were then quantified for 8 weeks (C). Numbers above each bar represent p-values for tumor sizes compared to controls.

To validate the traditional stochastic model of CD133^+^ MSCs, we derived two patient cell populations, either enriched for, or depleted of, cell surface CD133, and found that each population consistently maintained their CD133 status, since 97.2% and 95% of CD133^+^ cells expressed this marker in passage 2 (Fig. 3A) and 8 (Fig. 3B) as determined by flow cytometric analysis. Likewise, CD133^−^ cells retained their phenotype in culture for eight passages. Passage 8 CD133^+^ or CD 133^−^ cells were then xenotransplanted into immunodeficient mice. Successful xenotransplantation with as few as 1,000 CD133^+^ cells demonstrated their ability to repopulate and form tumors (p<0.03) in athymic *NCr-nu/nu* mice within a short span of 8 weeks. However, tumor formation could not be observed in the mice injected with matched cell density of CD133^−^ phenotype, nor with up to 200,000 cells (Fig. 4A and B).

## Discussion

This study has demonstrated, for the first time, that there exists a clonal dominance of the CD133 stemness marker within the hierarchy of cells in the cutaneous tissues from patients that have undergone successive progressive stages of melanoma, from primary to metastatic lesions. In addition, overexpression of CD133 correlates with the prognostic marker of tumor thickness in primary melanoma. A correlation also exists between the consistent overexpression of CD133 marker (p<0.02) and CD133 gene transcript levels in cell lines from metastatic patients with poor clinical outcome and short overall survival status. These findings suggest that CD133 represents a hyperpolarized cell population with tumorigenic potential at metastatic sites and is linked to survival status of the patient.

Although most CSCs in metastatic colon cancer (19), non-small cell lung carcinoma (20,21), glioblastoma (22), oral squamous cell carcinoma (23) and metastatic melanoma (7,14) have been detected as rare cell populations with tumorigenic capacity *in vitro* and *in vivo*, these studies have not been able to define a phenotypically distinct cell population with tumorigenic potential in target tissues with different clinical outcome or associated with the progression of the disease.

It has been widely reported that CSC represent rare tumor cell populations that possess unique properties making them important for tumor initiation, growth and metastasis. As per the CSC hypothesis, tumor initiation is regarded as an exclusive characteristic of CSCs (24). However, their distribution is highly variable depending on the clinical stage and anatomic site of the tumor. Although our previous study has demonstrated varied frequencies of expression of melanoma stem cell markers at different stages of the disease and site of the tumor tissue (10), our present study clearly delineates clonal dominance of CD133 in tissues of patients with primary melanoma who had disease progression or disease recurrence in comparison with primary melanoma with no recurrence, suggesting that the CD133 subset may be a determinant of tumor metastasis/tumor relapse. Our *in vivo* assessment of a CD133^+^ subset population further confirmed that CD133^+^ MSCs contain self-renewal and repopulating capabilities.

Although the CD133 cell population co-expresses other stem cell markers or may represent overlapping markers, recent reports on metastatic melanoma containing CD133 and ABCG2-positive cells with enhanced tumorigenic potential further support our present findings on consistent expression of stemness marker CD133 in conjunction with ABCB5 in various progressive stages of cutaneous melanoma (25,26). Further the proportionately lower incidence of CD133 positive tissues in distant metastatic melanomas that successively progressed from primaries, are possibly associated with selective pressure of a hypoxic niche or impact of novel mechanisms of dormancy of melanoma stem cells within the post-metastatic tissue micro-environment.

Our gene quantitation analysis of CD133 in cell lines from metastatic melanoma patients with poor clinical outcomes and short overall survival strongly supports our hypothesis that CD133 is a reliable marker of disease recurrence and an abundance of CD133 transcripts may play a pivotal role in progression of human cutaneous melanoma.

Based on our encouraging leads on clonal dominance of tumorigenic CD133^+^ phenotypes in tumor metastasis and tumor recurrence as determined by immunohistochemical expression in tissues from patients with short overall survival and poor clinical outcome, as well as gene expression analysis by qRT-PCR, we further validated our hypothesis by examining their *in vivo* tumorigenicity by utilizing xenotransplantation experiments in immunodeficient mice.

Our xenotransplantation studies with CD133^+^ cells in nude mice clearly demonstrated that as few as 1,000 CD133^+^ cells are capable of generating rapidly growing tumors in nude mice within a short period of 8 weeks thus demonstrate positive correlation between enrichment of more aggressive CD133^+^ phenotypes and tumor growth. This further reminds us that even CSCs are susceptible to selection pressure in aggressive tumors. This observation is a reflection of its pre-existence in the original metastatic melanoma prior to its resection from the patient. We believe it is likely that the metastatic phenotype existed in unrecognized form in the pre-metastatic lesions in the patients. Our findings sufficiently justify the basis of identifying patients whose cancers are at high-risk for metastases even in the absence of clinical evidence of metastases.

It is still not clear whether melanoma stem cells are derived from melanocytic stem cells, melanocytic progenitors, or mature melanocytes. It is assumed that primary CSCs originate through cell fusion with the cell population and that they have altered intrinsic/epigenetically unstable mechanisms with an enhanced metastatic potential and drug resistance. Further, the pathways and signaling molecules involved in stem cell homeostasis, de-differentiation, and transformation to melanoma stem cells remain to be determined. These molecules or pathways associated with melanoma stem cells would serve as potential candidates for targeted therapies.

Substantial variation exists on the precise definition of melanoma stem cells based on the traditional stochastic model. However, our results uniquely provided a rationale for the definition of a predictive factor of disease progression and represent a significant step towards identifying a more promising target in the clinical management of high-risk cutaneous melanoma.

## Figures and Tables

**Table I.** Immunoexpression of MSC markers in patients with or without disease recurrence.

Disease stage	Immunoexpression of MSC markers			
	CD133 (%)	ABCB5 (%)	CD166 (%)	Nestin (%)
Group I: no recurrence (n=18)	5/18 (27)	8/18 (44)	10/18 (55)	16/18 (88)
Group II: recurrence (n=18)	11/18^a^(61)	9/17 (52)	9/18 (50)	15/18 (83)

a p<0.02 (Fisher’s exact test); p<0.025 (Odds ratio analysis). Relative risk of CD133 overexpression = 2.2.

**Table II.** Immunoexpression of MSC markers vs. primary human melanoma thickness.

	Immunoexpression of melanoma stem cell markers			
Thickness of primary lesion	CD133 (%)	ABCB5 (%)	CD166 (%)	Nestin (%)
≤1.00 mm (n=8)	2/8 (25)	5/8 (62)	3/8 (37)	8/8 (100)
>1.00 mm (n=32)	16/32^a^(50)	15/31 (48)	18/32 (56)	27/32 (84)

a p>0.05.

**Table III.** Expression of MSC markers in patients with primary melanoma successively progressed to lymph node and distant organ metastasis.

	Immunoexpression of melanoma stem cell markers			
Disease stage	CD133 (%)	ABCB5 (%)	CD166 (%)	Nestin (%)
Primary melanoma (n=8)	8/8 (100)	8/8 (100)	3/8 (37)	8/8 (100)
Lymph node metastasis (n=8)	8/8 (100)	8/8 (100)	0/8 (0)	8/8 (100)
Distant organ metastasis (n=6)	5/6^a^(83)	4/6^a^(66)	2/6 (33)	6/6 (100)

a p>0.05.
